# A head-to-head comparison of TNF-α inhibitors as the first biologic treatment of rheumatoid arthritis

**DOI:** 10.1093/rheumatology/keag007

**Published:** 2026-01-14

**Authors:** Amir Aamodt Kazemi, Eirik Klami Kristianslund, Joseph Sexton, Karen Minde Fagerli, Sella Aarrestad Provan, Inge Christoffer Olsen

**Affiliations:** Oslo Centre for Biostatistics and Epidemiology, Institute of Basic Medical Sciences, Faculty of Medicine, University of Oslo, Oslo, Norway; Center for Treatment of Rheumatic and Musculoskeletal Diseases (REMEDY), Diakonhjemmet Hospital, Oslo, Norway; Department of Research Support for Clinical Trials, Oslo University Hospital, Oslo, Norway; Center for Treatment of Rheumatic and Musculoskeletal Diseases (REMEDY), Diakonhjemmet Hospital, Oslo, Norway; Center for Treatment of Rheumatic and Musculoskeletal Diseases (REMEDY), Diakonhjemmet Hospital, Oslo, Norway; Center for Treatment of Rheumatic and Musculoskeletal Diseases (REMEDY), Diakonhjemmet Hospital, Oslo, Norway; Center for Treatment of Rheumatic and Musculoskeletal Diseases (REMEDY), Diakonhjemmet Hospital, Oslo, Norway; Oslo Centre for Biostatistics and Epidemiology, Institute of Basic Medical Sciences, Faculty of Medicine, University of Oslo, Oslo, Norway; Department of Research Support for Clinical Trials, Oslo University Hospital, Oslo, Norway

**Keywords:** rheumatoid arthritis, TNF inhibitors, instrumental variable analysis, comparative efficacy

## Abstract

**Objectives:**

TNF-α inhibitors (TNFis) are effective biologic drugs for rheumatoid arthritis (RA), but their comparative efficacy is unclear due to limited direct comparisons. The objective of this work was to compare the efficacy of infliximab (INX), certolizumab pegol (CZP), etanercept (ETN), golimumab (GOM) and adalimumab (ADM) in achieving remission at 3 months in bio-naïve RA patients.

**Methods:**

Using data from the NOR-DMARD study of adult RA patients in Norway starting a TNFi as their first biologic treatment, we employed a target trial emulation approach. TNFi selection is mainly determined by drug prices in annual national tenders, allowing us to use drug cost as an instrumental variable (IV). Applying a novel IV method, causal effects were estimated with remission at 3 months as the end point. Results were compared with standard regression analysis adjusted for baseline variables.

**Results:**

The IV analysis suggested potential differences in efficacy among TNFis, with INX showing higher remission rates compared with CZP, and ETN performing better than GOM. However, confidence intervals were wide, and many comparisons were not statistically significant. Sensitivity analyses confirmed the overall direction of results. Regression analysis adjusting for observed confounders showed no substantial differences, underscoring the importance of addressing unobserved confounding effects.

**Conclusion:**

This hypothesis-generating study provides evidence that TNFis may not be equally effective. The uncertainty in the estimates highlights the need for a pragmatic randomized controlled trial to validate the findings. With decreasing costs of TNFis, such studies are increasingly feasible and critical to guide clinical decision-making.

*Rheumatology* key messages

- TNF-α inhibitors may not be equally effective; infliximab appears more efficacious than others.
- Findings challenge assumptions of equivalent efficacy, emphasizing need for pragmatic randomized trials to confirm.
- Addressing unobserved confounding is crucial; instrumental variable methods interpreted as target trials offer a solution.

## Introduction

Rheumatoid arthritis (RA) is a chronic inflammatory disease that significantly affects the quality of life of patients [1]. The introduction of TNF-α inhibitors (TNFis), a class of biologics, has greatly improved the management of this disease [2]. Although all TNFis target the same cytokine, they differ somewhat in their structure, relative dose and mechanism of action, which may have implications for their efficacy [3]. All TNFis have been proven to be effective for RA, but it is not a given that they are equally effective. This paper provides a head-to-head comparison of TNFis as first-line biologic treatments for RA.

### Background

Few randomized controlled trials (RCT) provide head-to-head comparisons of biologic treatments for RA [4]. The systematic reviews informing the most recent European recommendations identified 10 head-to-head trials of biologic drugs, but only one that included two TNFis [2, 5, 6]. This RCT (EXXELERATE) did not find differences between adalimumab (ADM) and certolizumab pegol (CZP) in the proportion of patients reaching response as defined by the American College of Rheumatology (ACR20) or a state of low disease activity score at week 12 or 104 [2].

In the absence of RCTs, researchers have resorted to observational studies and network meta-analyses to compare the effectiveness of TNFis. A recent systematic review identified 14 observational studies comparing the effectiveness of TNFis, without any evident differences reported [7]. However, this result comes with caution, as none of the studies are considered at low risk of bias and only two are considered at moderate risk.

Due to this knowledge gap, TNFis are often considered equally effective in treatment guidelines. However, individual factors, for example, co-morbidities and preferred administration route, can influence drug choice. Further background is provided in Supplementary Appendix Subsection C.1.

### Novelty

The Norwegian Drug Procurement Cooperation (LIS) [8] has a yearly tender for biologic treatments, including TNFis. Prescribing physicians are instructed to initiate treatments with the least expensive alternative unless there are strong reasons to select a different TNFi. Kazemi and Olsen [9] have recently developed a new instrumental variable (IV) methodology to compare multiple interventions. IV analysis estimates treatment effects using variables that influence treatment choice but not the outcome directly, emulating an RCT and reducing bias. The LIS recommendation in Norway can serve as an IV to compare the real-life efficacy of TNFis. Our previous work shows the feasibility of this approach. Here, we present a complete comparative analysis of TNFis as first-line biologic treatments for RA, using the IV method in a target trial emulation approach, providing both methodological and clinical discussions.

### Outline

In ‘Methods’ we provide descriptions of our data and methods. In ‘Results’ we present the result of our analyses in addition to descriptive statistics of our data set. In ‘Discussion’ we provide detailed discussions of the results, utilizing a target trial emulation approach.

## Methods

To estimate causal effects, the categorical IV estimator, developed by Kazemi and Olsen [9], was applied to data from the NOR-DMARD study, taking advantage of the history of LIS recommendations as an instrument. In addition, a logistic regression model was fitted to the outcome and marginalized as a conventional comparator method. This is better known as standardization in the causal inference literature [10, 11]. A mathematical formulation of the methods is provided in Supplementary Appendix Subsection A.5. In addition to the main analysis, we performed subgroup analyses stratifying on age group and seropositivity, the results of which are presented in Supplementary Appendix Subsection B.6. Ethical approval for the NOR-DMARD project, which provided the data for this paper, was obtained from the Regional Committees for Medical and Health Research Ethics (REK). Written informed consent to participate was obtained from all patients included in NOR-DMARD. This paper includes no identifying information about the participants.

### Data

The NOR-DMARD study includes patients with inflammatory arthritis initiating a disease-modifying antirheumatic drug (DMARD), such as a TNFi, in a clinical setting. It tracks treatment courses and outcomes at preplanned regular visits. We used data from this study in the period 2010–2023, when producers of all five TNFis participated in the LIS tender. The population was limited to adult patients with RA who initiated a biologic treatment for the first time (bio-naïve patients). This ensured that TNFis were the only relevant treatment alternatives in line with national and international guidelines on RA treatment. Note that these patients were also naïve to targeted synthetic DMARDs.

Clinical remission 3 months after starting treatment was our primary outcome. This was the first decision time point according to the treat-to-target principle [12]. Remission was derived from the disease activity score (DAS28 CRP <2.6). This is a composite score encompassing both clinical and patient-reported outcomes. The primary target parameter was the difference in the average probability of remission. Missing data were imputed with multiple imputations with chained equations (MICE) with *m* = 10. A sensitivity analysis with single imputation based on termination date is presented in Supplementary Appendix Subsection B.5. Further description of our handling of missing data is provided in Supplementary Appendix Subsection A.1.

Treatment was a categorical variable with five levels: infliximab (INX), etanercept (ETN), golimumab (GOM), CZP and ADM, further described in Supplementary Appendix Subsection 6.2.

### Regression analysis

To address measured confounding in the regression analysis, we adjusted for baseline variables available in the NOR-DMARD study with fewer than 70% missing values, with no interaction terms. Key covariates are summarized in Table 1, while the complete list is provided in Supplementary Appendix Subsections A.3 and B.1. Fig. 1 presents a directed acyclic graph showing the assumed causal structure with the covariates grouped into five categories. In Supplementary Appendix Subsection C.3, we further justify this choice of comparator method.

**Figure 1:**
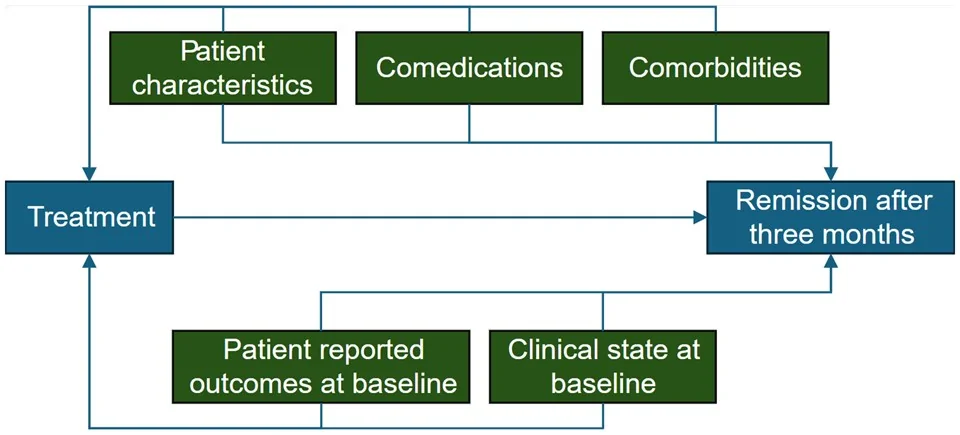
Directed acyclic graph showing the assumed causal structure in the regression analysis with the covariates grouped into five categories. The green boxes represent observed confounding variables and this graph assumes no confounding effects beyond these. Treatment is a categorical variable with five levels indicating one of the five TNF-α inhibitors. Remission at 3 months is a binary variable, while the confounding variables can be a mixture of categorical, binary and continuous baseline variables

**Table 1:** Summary of data.

	Treatment					
	ADM (*n* = 432)	CZP (*n* = 295)	ETN (*n* = 472)	GOM (*n* = 65)	INX (*n* = 182)	All (*n* = 1446)
Remission at 3 months, %	49 [38]	45 [14]	47 [25]	41 [22]	45 [13]	46 [25]
Age, mean (s.d.), years	52 (15) [0]	55 (14) [0]	51 (15) [0]	50 (13) [0]	55 (14) [0]	53 (15) [0]
Male, %	26 [3]	30 [1]	24 [1]	30 [2]	30 [3]	27 [2]
Smoker current, %	14 [42]	17 [30]	16 [28]	22 [8]	22 [37]	17 [33]
Smoker occasionally, %	0 [42]	1 [30]	2 [28]	5 [8]	2 [37]	2 [33]
Smoker previously, %	40 [42]	38 [30]	42 [28]	28 [8]	40 [37]	40 [33]
RF positive, %	43 [38]	55 [42]	66 [19]	75 [18]	51 [45]	57 [33]
Anti-CCP positive, %	61 [27]	70 [31]	75 [13]	76 [15]	70 [32]	70 [24]
Previous MTX user, %	90 [4]	86 [3]	75 [4]	72 [0]	89 [4]	83 [3]
Current MTX user, %	75 [0]	79 [0]	55 [0]	83 [0]	75 [0]	70 [0]
Current Prednisolone user, %	24 [0]	53 [0]	45 [0]	60 [0]	42 [0]	41 [0]
Current Sulfasalazine user, %	7 [0]	14 [0]	9 [0]	9 [0]	8 [0]	9 [0]
Current user of other DMARDs, %	2 [0]	2 [0]	4 [0]	2 [0]	3 [0]	3 [0]
PGA (0–100), mean (s.d.)	43 (27) [14]	46 (25) [7]	44 (26) [8]	46 (24) [2]	46 (27) [13]	44 (26) [10]
PhGA (0–100), mean (s.d.)	29 (17) [29]	35 (17) [29]	31 (17) [14]	35 (17) [12]	32 (18) [23]	32 (17) [22]
ESR, mean (s.d.), mm/h	18 (17) [29]	20 (17) [26]	20 (16) [18]	20 (17) [22]	21 (18) [19]	20 (17) [23]
CRP, mean (s.d.), mg/L	10 (16) [15]	11 (16) [11]	9 (16) [10]	13 (18) [8]	11 (17) [12]	10 (16) [12]
SJC28, mean (s.d.)	3 (4) [16]	4 (4) [13]	4 (4) [8]	4 (4) [5]	5 (5) [14]	4 (4) [12]
TJC28, mean (s.d.)	5 (5) [16]	5 (5) [13]	5 (5) [7]	6 (7) [5]	6 (6) [14]	5 (5) [12]
DAS28 CRP, mean (s.d.)	3.5 (1.3) [23]	3.8 (1.2) [19]	3.7 (1.2) [15]	4.0 (1.3) [9]	3.8 (1.4) [19]	3.7 (1.3) [18]
Pain (0–100), mean (s.d.)	37 (26) [22]	42 (24) [20]	40 (25) [48]	55 (25) [75]	42 (26) [35]	40 (25) [34]
Joint pain (0–100), mean (s.d.)	37 (26) [36]	43 (25) [7]	40 (25) [11]	46 (27) [2]	42 (27) [15]	41 (26) [18]
EQ index, mean (s.d.)	1 (0) [4]	1 (0) [4]	1 (0) [4]	1 (0) [2]	1 (0) [8]	1 (0) [5]
MHAQ score, mean (s.d.)	0 (0) [14]	1 (0) [7]	1 (0) [8]	1 (0) [2]	1 (0) [14]	1 (0) [10]
RAID score, mean (s.d.)	4 (2) [43]	4 (2) [33]	4 (2) [53]	5 (2) [78]	4 (2) [45]	4 (2) [46]
Employment, %	65 [68]	57 [39]	55 [66]	60 [85]	51 [46]	57 [59]
Diagnosis duration, mean (s.d.), years	6 (7) [41]	9 (10) [25]	7 (8) [28]	7 (7) [12]	7 (10) [30]	7 (9) [30]
Symptom duration, mean (s.d.), years	7 (7) [70]	9 (8) [55]	8 (7) [69]	13 (8) [78]	9 (12) [73]	8 (8) [67]

All variables are measured at baseline unless otherwise specified. Values in square brackets are percentage of missing data. CCP: cyclic citrullinated peptide; CRP: C-reactive protein; DAS: disease activity score; DMARD: disease-modifying antirheumatic drug; EQ: EuroQol; ESR: erythrocyte sedimentation rate; MHAQ: modified health assessment questionnaire; MTX: methotrexate; PGA: patient global assessment; PhGA: physician global assessment; RAID: rheumatoid arthritis impact of disease; RF: rheumatoid factor; SJC: swollen joint count; TJC: tender joint count.

### Instrumental variable analysis

In the IV analysis, we used an exogenous variable (the instrument), affecting the treatment choice but not the outcome, to address unobserved confounding effects. The price-ordered list of the treatment alternatives, with a large effect on the treatment choice, served as the instrument. The exogeneity of this variable is discussed in Supplementary Appendix Subsection C.2.

The IV analysis relies on the concept of an adherence set, *A*, defined as the set of treatment alternatives with a non-zero probability of being chosen by a decision team; i.e. for a physician–patient pair, *A* is the set of relevant treatment alternatives [9]. To improve identifiability and interpretability, adherence sets were restricted to 14 clinically justifiable sets, listed in Supplementary Appendix Subsection A.4. We performed a sensitivity analysis allowing all possible adherence sets, in addition to a secondary analysis optimized for identifiability. We assumed that decision teams always choose the least expensive drug in their adherence set. The directed acyclic graph in Fig. 2 illustrates the assumed causal structure in the IV analysis. Similar to baseline table evaluation in RCT reports, we evaluated the generalizability of the results by comparing patient characteristics across treatment groups within the target population.

**Figure 2:**
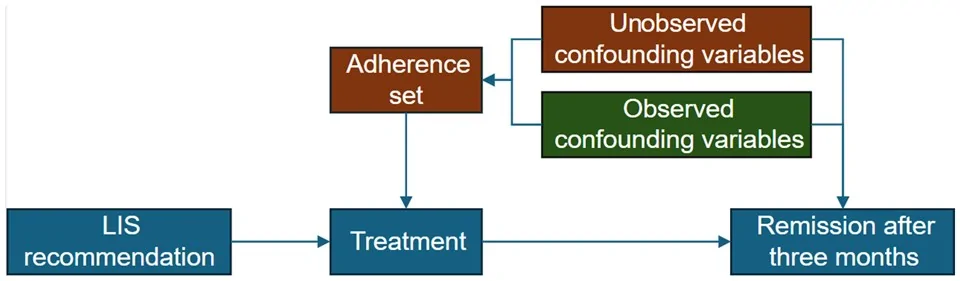
Directed acyclic graph illustrating the assumed causal structure in the IV analysis. The unobserved variables are shown in red boxes. The Norwegian Drug Procurement Cooperation (LIS) recommendation is an ordered list of the five TNF-α inhibitors (TNFis). Treatment is a categorical variable with five levels indicating one of the five TNFis. Adherence set is a categorical variable with 14 levels listed in Supplementary Appendix Subsection A.4. Remission at 3 months is a binary variable, while the confounding variables can be a mixture of categorical, binary and continuous baseline variables

### Target parameters

In the regression analysis, positivity was assumed, meaning all treatment alternatives have a non-zero probability of being prescribed given any values for the confounding factors. Under positivity, the target parameter was the average treatment effect (ATE), meaning the target population was the entire adult bio-naïve RA population in Norway.

In the IV analysis, the positivity assumption was relaxed. A patient–physician pair may have any adherence set listed in Supplementary Appendix Subsection A.4. Positivity was assumed only for the treatment alternatives in their adherence set. Local average treatment effects (LATEs) were then identified for adherence set groups, meaning the target population was adult bio-naïve RA patients in Norway whose adherence sets satisfy specific comparison conditions.

With five treatments, 10 head-to-head comparisons are possible. Regression analysis provides estimates for all 10 effects. However, only five were reliably identifiable in the IV analysis (with $\hat{P}(A\in \Pi_{t-t'})>0.1$), shown in Fig. 3, four of which were also identified in the sensitivity analysis allowing all adherence sets, presented in Supplementary Appendix Subsection B.3. In Supplementary Appendix Subsection B.2, we present a secondary analysis identifying two additional effects involving ADM.

**Figure 3:**
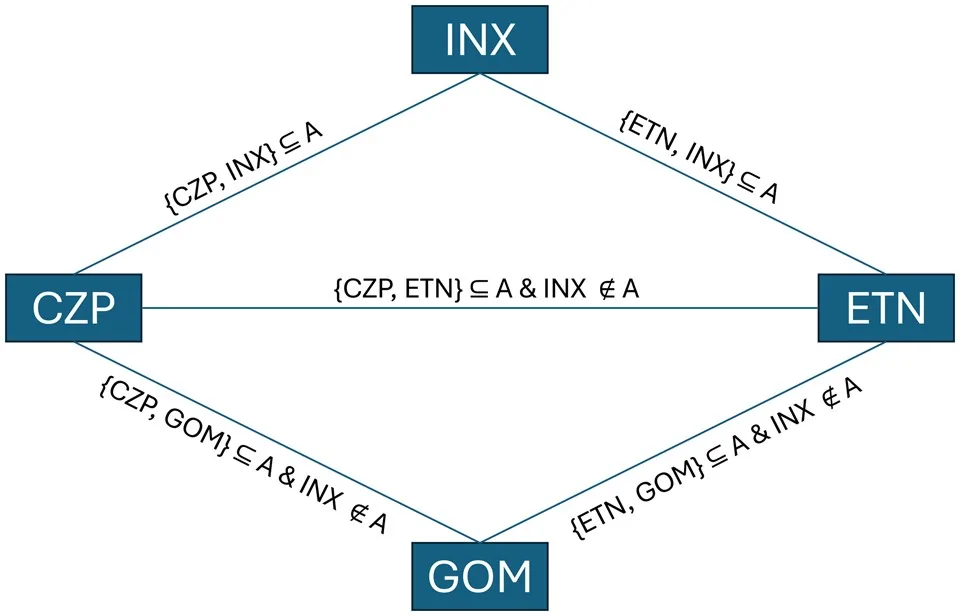
A presentation of the head-to-head comparisons in the instrumental variable analysis. An edge between two treatments means an identifiable effect. On each edge the condition of the identifiable LATE is provided ($\Pi_{t-t\prime })$. The conditions are on the adherence sets (A) of the decision teams in the target populations. A drug belongs the adherence set of a decision team if and only if that decision team has a non-zero probability of choosing that drug. CZP: certolizumab pegol; ETN: etanercept; GOM: golimumab; INX: infliximab

## Results

Table 1 summarizes treatment data, outcomes, and key baseline variables. Fig. 4 presents the LIS tender results, as well as the number of patients, remission rates and treatment probabilities under each LIS recommendation. Comorbidity data are summarized in Supplementary Appendix Table B1.

**Figure 4:**
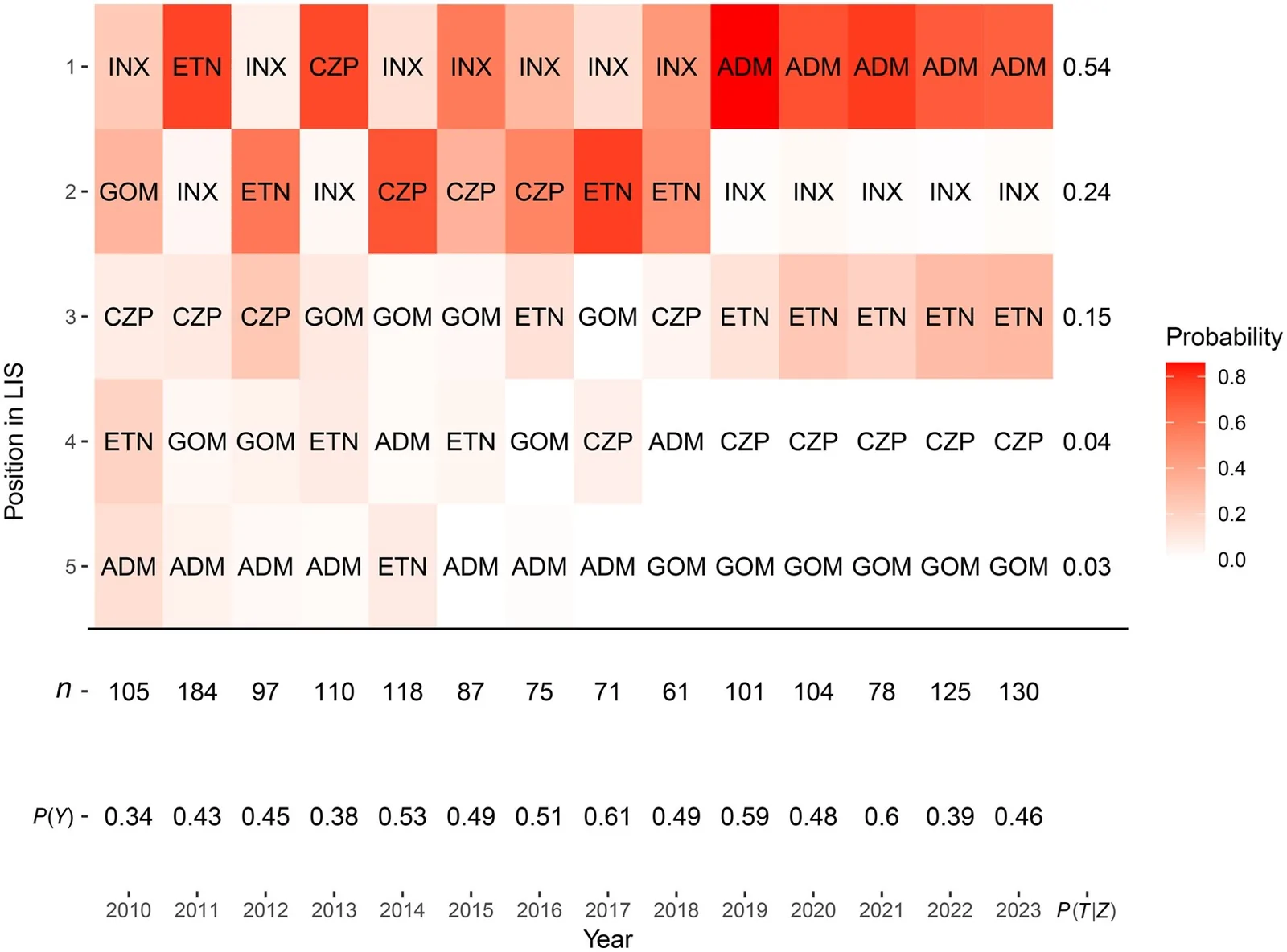
LIS tender results and the number of patients, remission rates and treatment probabilities under each LIS recommendation. Each column shows a tender and the rows show the LIS recommendation. The *y*-axis is the place in the price list, where the first row is the most recommended (least expensive) drug for first line of treatment. The last two rows show the number of bio-naïve patients (*n*) in NOR-DMARD that receive their first TNFi in the period corresponding to that LIS recommendation and the remission rate [*P*(*Y*)] for these patients at 3 months after treatment start. The colour indicates the proportion of these patients who receive the corresponding drug. The last column, P(T|Z), shows the overall probability of receiving the corresponding drug in the whole study period. ADM: adalimumab; CZP: certolizumab pegol; ETN: etanercept; GOM: golimumab; INX: infliximab; LIS: Norwegian Drug Procurement Cooperation

GOM was the least prescribed drug during the study period, as it never won the LIS tender. INX was the second least prescribed despite winning the tender in 7 out of 14 years, possibly due to its unavailability for subcutaneous administration. Otherwise, the heat map in Fig. 4 supports high relevance of the LIS recommendation for the prescribed treatment, showing the highest probabilities for the drugs ranked first or second. Plausible reasons to deviate from the LIS recommendation are further discussed in Supplementary Appendix Subsection A.4.

Fig. 5 shows the effect estimates from both analyses. The primary IV analysis indicated the inferiority of GOM and the superiority of INX to CZP and ETN, while it detected no difference between CZP and ETN. The sensitivity analyses confirmed this result. The stratified secondary analyses indicated that the superiority of INX over ETN was strongest for the younger and seropositive patients. The secondary IV analysis providing comparisons involving ADM suggested no efficacy difference between ADM and CZP or ETN. All confidence intervals from the regression analysis contained the null, with no indication of substantial efficacy differences between the drugs.

**Figure 5:**
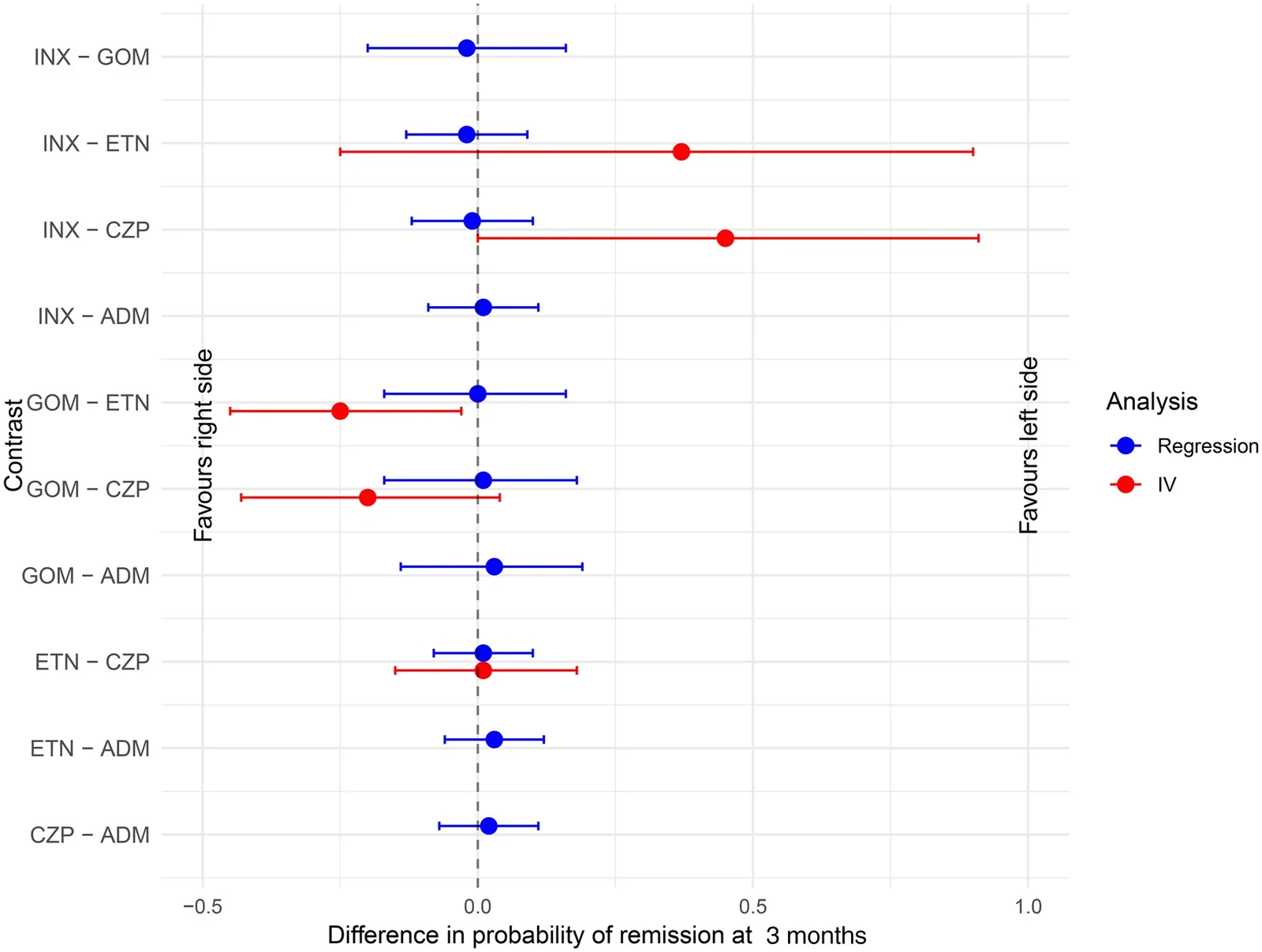
Effect estimates from both analyses with confidence intervals. For the regression analysis the CIs are derived analytically from the model while for the IV analysis the CIs are calculated using bootstrapping with 1000 replications, excluding the replications that yield an estimate outside the [−1, 1] interval. In both cases, the uncertainty is calculated for each imputed data set and then pooled. Note that for each contrast, *t*_1_ − *t*_2_, the *x*-axis is $E(Y^{T=t_{1}}-Y^{T=t_{2}})$. Therefore, an estimate on the right side of the dashed black line means that the left side of the contrast *t*_1_ − *t*_2_ has better efficacy, and vice versa. ADM: adalimumab; CZP: certolizumab pegol; ETN: etanercept; GOM: golimumab; INX: infliximab; IV: instrumental variable

## Discussion

With a novel approach to a unique dataset, we estimated varied efficacy among TNFis for bio-naïve RA patients. Remission rates 3 months after treatment start were higher for INX compared with CZP, and for ETN compared with GOM. In this hypothesis-generating study, the analysis was not adjusted for multiple testing. The superiority of INX over CZP is valid only when all TNFis are considered potential treatment alternatives, while ETN’s superiority over GOM is valid only when intravenous injection is excluded as a relevant alternative.

INX, administered intravenously with induction dosing, may have superior results after 3 months to subcutaneously administered CZP. In addition, INX has been shown effective in other diseases, including ulcerative colitis and Crohn’s disease, indicating a broad-acting mechanism of action. ETN and GOM differ in their dosing schedule, with ETN dosed weekly and GOM monthly, possibly affecting the speed with which their effects are realized.

ADM was not part of our primary analysis due to low instrument variation. Our secondary analysis did not identify any differences in the efficacy of ADM compared with CZP or ETN, aligning with the EXXELERATE study. Otherwise, our findings contrast the previous assumption and observational findings of no difference between TNFis. However, prior observational studies, at high risk of confounding, lack unbiased methodology.

To generalize the results of our IV analysis to the entire patient population, one would need to assume that treatment effects are the same across all patient groups (i.e. no effect modification). In practical terms, this means that although individual patients may respond differently to different TNFis, these differences are not known in advance and therefore do not influence treatment choice. This is a strong assumption that may not hold. However, our aim is not to recommend that all patients should be forced onto one specific TNFi. Therefore, the generalization to the entire patient population is not needed. As discussed below (‘IV analysis’), our head-to-head comparison results are only valid in cases where both treatment alternatives being compared are viable options. The proposed intervention could then be that central health authorities influence prescribing patterns through guidelines based on comparative efficacy research, as they already do based on drug prices. In this context, one could argue that the LATEs identified by IV analysis are the most relevant quantities, since they reflect the treatment effects among the decision teams whose choices are actually influenced by such policies [13].

The IV and regression analyses yielded different results. This deviation could possibly be explained by estimation error, unobserved confounding effects, positivity violations and/or effect heterogeneity. Despite substantial differences in the point estimates, the overlapping confidence intervals for all effects identified by both analyses mean the deviations could be explained by random chance. However, systematic method differences might also exist. Unobserved confounding effects or positivity violations might have resulted in biases in the regression analysis, making a comparison to the unbiased IV estimates inappropriate. In the section ‘Regression analysis’, we discuss the plausibility of such violations of the assumptions in the regression analysis. Additionally, if the effects identified by the IV analysis differ in the local populations from the ATE, the estimates are expected to be different in the two analyses. In the section ‘IV analysis’, we discuss the conditions under which the IV estimates are valid. Table 1 shows that a substantial percentage (25%) of all included patients have missing outcome. While our method of handling missing data, presented in Supplementary Appendix Subsection A.1, is shown to be robust [14–17], a violation of the missing at random assumption underlying our method could introduce biases into the analyses. However, results from our sensitivity analysis, presented in Supplementary Appendix Subsection B.5, in addition to results from earlier literature [9] support the validity of this assumption.

### Regression analysis

To estimate the ATE, the regression analysis assumes positivity, meaning all patient subgroups can receive any treatment. While no covariate combination in NORDMARD clearly excludes a TNFi, many patients receive more expensive treatments than recommended. Therefore, there must be strong reasons for not prescribing the least expensive drug to these patients, possibly violating positivity. To address this issue, the target population should be modified, limiting the analysis only to those patients for whom positivity holds. However, most of the factors that are likely candidates for this stratification, such as distance to hospital or physician’s preferences, are unobserved. In addition to creating an estimation bias, this weakness results in a challenge in interpreting for whom the effect estimates coming from the regression analysis are valid.

Another source of bias in the regression analysis could be unobserved confounding effects. In Supplementary Appendix Subsection C.4, we provide plausible examples of such effects.

If unobserved confounding effects are present, the results in Fig. 5 indicate a net negative confounding effect (towards the null), possibly explaining previous observational results. This is reasonable since if a drug in fact is more efficacious and the physicians are to some degree aware of that effect, that drug is more likely to be prescribed to the least healthy patients. These patients have the lowest probability of achieving remission, creating a negative confounding effect.

### IV analysis

Fundamental to the interpretation of the IV analysis is the concept of an adherence set, which can be likened to a list of relevant treatments for a patient provided by the physician based on factors that are possibly unobserved in our data set. The IV analysis emulates platform trials with multiple treatments, where eligibility affects the randomization and is adjusted for in the analysis, as seen in trials implemented to identify treatments for COVID-19 [18, 19].

If one considers each link in Fig. 3 as a comparison between the two treatments that it connects, the IV results can be discussed as a target trial, where a drug’s presence in the adherence set serves as an eligibility criterion for the corresponding arm. Consider the following pragmatic trial:

- Upon screening, the physician is asked to write down the list of relevant alternatives for the patient. This list can be based on observed or unobserved variables, such as planned pregnancy, work status, physician’s preferences, etc. Call this list the adherence set (*A*).
- Assign patients to receive one of the treatments in their adherence set at random and measure remission 3 months after treatment start.
- When analysing the results, condition each head-to-head comparison on the adherence set, applying the conditions given on each edge in Fig. 3.

Assuming exogeneity and that the only possible adherence sets are those listed in Supplementary Appendix Subsection A.4, the IV analysis estimates the result of this trial. The estimated summary of the most important baseline variables for patients included in each comparison is given in Supplementary Appendix Subsection B.7.

The INX–CZP comparison includes all decision teams that adhere to both INX and CZP. However, the only plausible adherence set including both of these treatments is full compliers, i.e. decision teams that have no reason to exclude any drugs. Decision teams that strongly prefer subcutaneous injection, humane antibodies, or the oldest TNFis, as well as patients with co-morbidities that exclude one or more alternatives, are all excluded from this comparison.

The INX–ETN comparison includes all decision teams who adhere to both treatments. This is a more inclusive analysis than INX–CZP because it, for example, includes cases where the physician has a strong preference for the oldest TNFis. Although the confidence interval is very wide and contains the null, the point estimate indicates INX’s superiority over ETN.

The ETN–CZP, GOM–ETN and GOM–CZP effects are estimated for the adherence sets including the drugs involved in the comparison and excluding INX. A plausible reason for excluding INX from *A* is preference for subcutaneous administration. This is likely for younger, fully employed patients or patients living far from the hospital. INX might also be ruled out to avoid high risk of antibody formation. For ETN–CZP and GOM–ETN the only plausible adherence set including the drugs compared also includes the other subcutaneously administered drugs. However, the GOM–CZP comparison is more inclusive than the other two, as adherence sets excluding ETN, e.g. due to preference for humane antibodies or rare dosing, are included in this population.

The comparison of Tables 1 and Supplementary Appendix Table B7 shows similar values for the baseline variables between the patients included in the analysis population of ETN–CZP and INX–CZP and the entire data set, supporting the generalizability of the result. However, for GOM–ETN and GOM–CZP, this comparison shows higher levels of smoking and RF positivity in the GOM arm than in the general population, which indicates that these results might be relevant for a smaller subset of the population.

The estimated summary of baseline variables, presented in Supplementary Appendix Subsection B.7, shows that there might be substantial differences in the baseline variables between the arms, possibly partially explaining some of the results. However, an adjusted IV analysis for baseline variables, presented in Supplementary Appendix Subsection B.4, returned results similar to the primary IV analysis.

## Conclusion

This hypothesis-generating study, utilizing a novel methodological approach based on causal inference techniques, suggests potential differences in efficacy among the TNFi agents studied. The results are consistent with INX being more efficacious, followed by ADM, ETN and CZP, while GOM may be less efficacious. However, the wide confidence intervals and exploratory nature of the analysis mean that these findings cannot be interpreted as confirmatory evidence. Differences in efficacy are likely influenced by dosing regimens and administration frequency. Using an IV approach and target trial emulation, our analysis overcomes key limitations of previous observational studies, providing a more robust comparison of TNFis in real-world clinical practice.

Our findings have important implications for treatment guidelines and clinical decision-making. With TNFis now comparably priced, these results challenge the long-standing assumption of equivalent efficacy among agents and underscore the need for personalized treatment strategies. However, the uncertainty in our estimates highlights the need for a pragmatic RCT to confirm these findings. Given the increasing feasibility of such trials, future research should aim to validate our results across diverse patient populations and longer follow-up periods to assess sustained clinical benefits.

Our approach could also be adapted to other health care systems where treatment choice is influenced by centralized procurement policies, formulary restrictions, or insurance reimbursement schemes. For example, national tenders in Denmark, recommendations made by the National Institute for Health and Care Excellence in the UK, or formulary-driven prescribing in Canada and the USA may provide similar sources of exogenous variation in treatment choice. In such settings, policy-driven variation can serve as a natural instrument, enabling IV analyses similar to those conducted here. The feasibility will depend on the degree to which such policies are binding in practice and the availability of high-quality longitudinal registry data.

This study demonstrates the potential of advanced causal inference methodologies in comparative effectiveness research, providing a framework for improving evidence-based decision-making in rheumatology. By integrating real-world data with rigorous statistical techniques, future research can further refine treatment strategies and optimize outcomes for patients with RA.

## Supplementary Material

keag007_Supplementary_Data

## Data Availability

All code, anonymous data from the NOR-DMARD registry and further information are available in the Open Science Framework (OSF) repository at: https://osf.io/dtcgh.
