# Supplementary material for: A head-to-head comparison of TNF-α inhibitors as the first biologic treatment of rheumatoid arthritis
Source: Rheumatology (Oxford). 2026 Jan 14;65(2):keag007. doi: 10.1093/rheumatology/keag007 (PMC13677782; doi:10.1093/rheumatology/keag007)
Supplement: Supplementary file 1 [file keag007_supplementary_data.pdf]

Appendix A Further Descriptions of the Data and the Methods

A.1 Handling of Missing Data

The outcome, clinical remission at three months after treatment start, is derived from the composite score DAS28 CRP. Measuring the outcome at three months allows enough time for the medications to take effect and limits the amount of missingness due to loss to follow-up. For the three-month visit, we use the closest visit to three months within the first six months of the treatment course. The outcome is considered missing if there are no such visits or if one of the components in DAS28 CRP is missing at that visit.

The outcome is missing for 550 of the 1446 patients included in the data set. This variable could be missing for several reasons. For instance, one of the components (TJC28, SJC28, PGA or CRP) could be missing. Another reason could be that there is no follow-up visit within the first six months of treatment start. In the case of missing DAS28 CRP, clinical remission is derived from DAS28 ESR, clinical disease activity index (CDAI) or termination reason. This is illustrated in Figure A1. We use 2.6 and 2.8 as cutoffs for DAS28 ESR and CDAI, respectively. Once this derivation is performed, 363 patients still have missing outcome. For these patients, the outcome is imputed with a logistic regression including all baseline variables (MICE with $m = 10$). A similar method is applied to impute missing values in baseline variables [20]. A sensitivity analysis is performed, where for the 519 patients still having missing outcome after imputation with DAS28 ESR and CDAI, a single imputation is made based on termination date, such that if the treatment is terminated within one year, the outcome is imputed as non-remission, and otherwise it is imputed as remission. The result of this sensitivity analysis is presented in Subsection B.5.

To calculate the uncertainty in the estimates, the uncertainty was estimated in each imputed data set and pooled. For the IV analysis, bootstrapping was used to estimate the uncertainty and for the regression analysis, uncertainty was extracted from the model. Note that the missing at random assumption underlying such imputation methods might have been violated in our study, resulting in biased estimators. While this is an untestable assumption, the agreement of the results of the sensitivity analysis presented in Subsection B.5 with the primary analysis supports the validity of this assumption, Additionally, Kazemi and Olsen [9] apply a different approach, imputing early terminations as non-remission and otherwise removing patients with missing outcome. They report significant superiority of CZP over ETN while not identifying the superiority of ETN over GOM. Their results otherwise agree with the results presented in this paper.

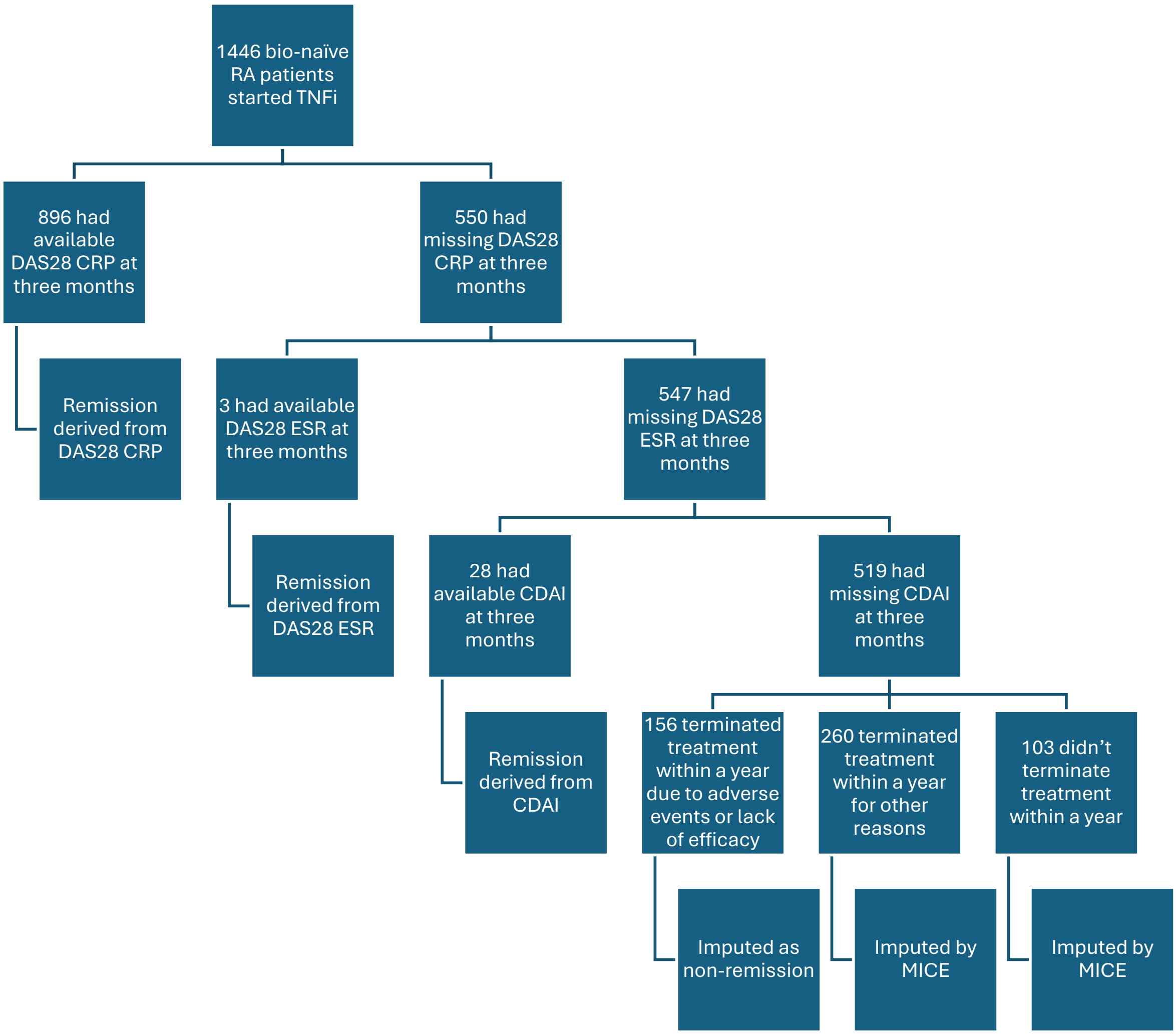

Fig. A1 Description of how the target variable (Remission at three months) is derived from clinical variables and how missingness in those variables is handled. Abbreviations: TNFi: TNF-α inhibitor, DAS: disease activity score, CRP: C-reactive protein, ESR: erythrocyte sedimentation rate, CDAI: clinical disease activity index, MICE: multiple imputation with chained equations. **Alt text:** This figure shows how missing outcome has been imputed taking advantage of alternative clinical scores, termination date and multiple imputation.

A.2 Handling of biosimilars

The treatment variable in our analysis represents the active substance in the drugs, some of which are off-patent and available from multiple producers. However, the large-molecule structure of the original drug is impossible to fully copy. Therefore, generic drugs are referred to as biosimilars. All biosimilars are grouped with their original drug. Treatment compliance is not measured. All effect estimates are therefore of treatment policy estimands.

A.3 Covariate List

The regression analysis is adjusted for 50 baseline variables, two of which, namely center and smoking status, are factor variables coded as five and two binary variables, respectively. In total, 56 variables are included, 21 of which are binary variables indicating co-morbidities at baseline. The co-morbidity variables are listed in Subsection B.1. The remaining covariates are listed and described as the following:

1. Center, i.e. the hospital where the treatment course is registered. Possible values are Diakonhjemmet, Drammen, Foerde, Lillehammer, St. Olavs and Tromsoe. Coded as five binary variables.
2. Patient's biological sex.
3. Patient's age at baseline.
4. Patient's smoking habit at baseline. Possible values are non-smoker, previous smoker and occasional smoker. Coded as two binary variables.
5. Rhematoid factor positivity.
6. Anti-CCP positivity.
7. Whether methotrexate was ever prescribed to the patient prior to baseline.
8. Whether methotrexate is prescribed as co-medication in the current course.
9. Whether prednisolone is prescribed as co-medication in the current course.
10. Whether sulfasalazine is prescribed as co-medication in the current course.
11. Whether other DMARDs than the three above are prescribed as co-medication in the current course.
12. Patient's global assessment of disease activity at baseline (0-100).
13. Physician's global assessment of disease activity at baseline (0-100).
14. Erythrocyte sedimentation rate measured at baseline
15. C-reactive protein level measured at baseline
16. Swollen joint count of 28 joints at baseline
17. Tender joint count of 28 joints at baseline
18. Swollen joint count of 32 joints at baseline
19. Tender joint count of 32 joints at baseline
20. ACREULAR remission at baseline
21. EuroQol5D index at baseline
22. Patient reported pain at baseline (0-100)
23. Patient reported joint pain at baseline (0-100)
24. Modified health assessment questionnaire score at baseline
25. Rheumatoid arthritis impact of disease score at baseline
26. Patient reported employment status at baseline (WPAI 1)
27. Patient reported degree of illness that affects regular activities at baseline (WPAI 6)
28. Diagnosis duration at baseline
29. Symptom duration at baseline

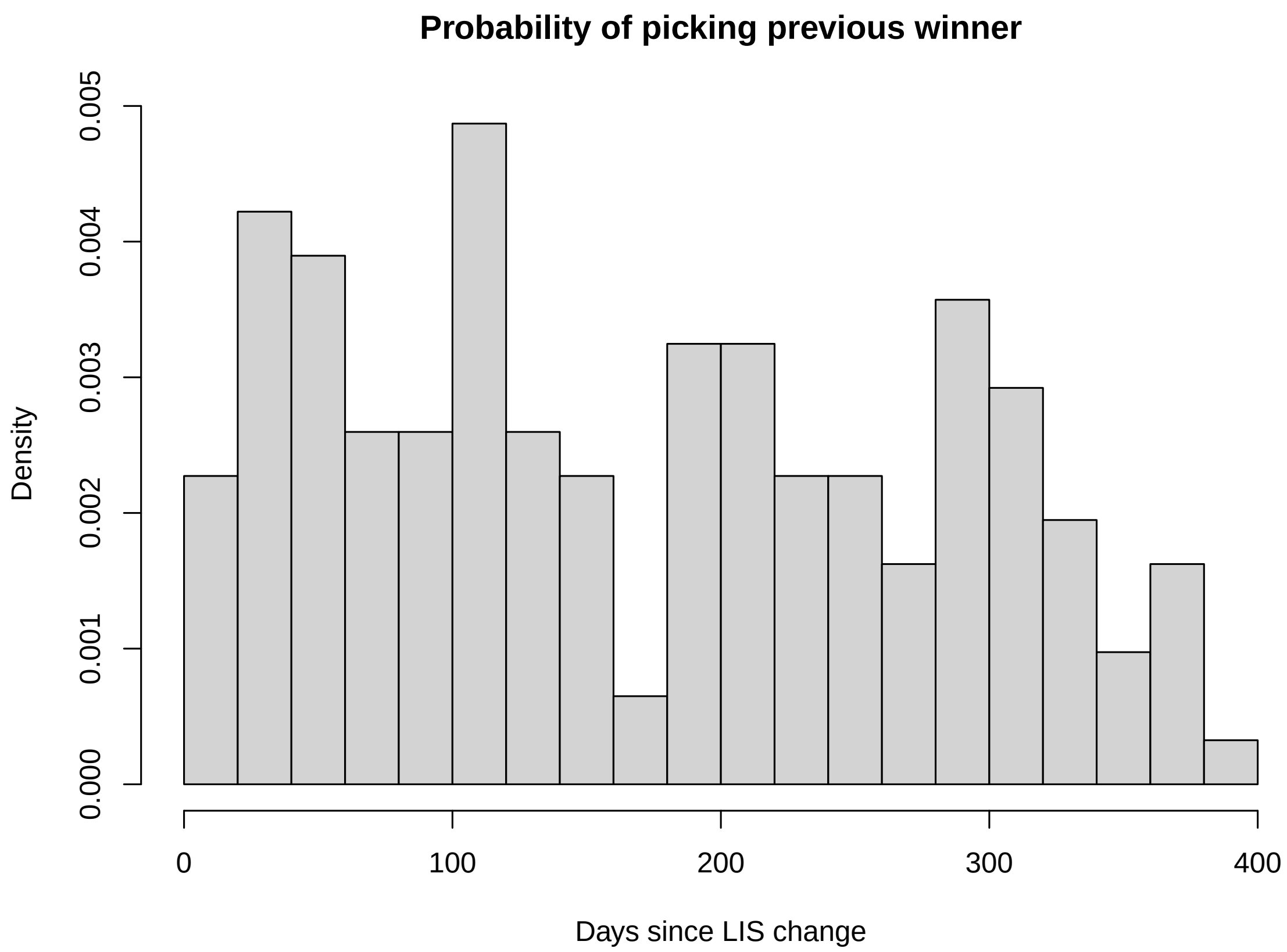

Fig. A2 A histogram showing the probability of the previous most recommended drug that is not the current most recommended, namely $\sum_{y=2}^{14} P(T = z_{y-1}[1] \,\&\, T \neq z_y[1] | Z = z_y)$. **Alt text:** This figure shows the probability of the previous most recommended drug as a function of days since a change in the LIS recommendation.

A.4 Adherence Set List

Although Figure 4 shows high relevance of the instrument, one can see substantial deviation in prescriptions from the most recommended drug. We argue that this is due to the existence of adherence sets other than full compliers. However, one could suspect that some of this deviation might be explained by a delay in response to changes in the LIS recommendation, i.e. physicians still prescribing the previous most recommended drug in the period immediately after a new recommendation is made. If this is the case, it is expected that the previous winner of the LIS tender is prescribed more commonly in the weeks immediately following the publication of the new LIS recommendation. In Figure A2 this probability is estimated with a histogram, and this figure shows a uniform distribution, which refutes this explanation.

The primary IV analysis is restricted to a set of 14 plausible adherence sets. This list was constructed in clinical discussions where a set was added to the list if we were able to find any clinical justification for it. This is done to improve identifiability and interpretability of the results. Note that the health

authorities in Norway require clinicians to follow the LIS recommendation unless there are strong clinical reasons to deviate from it. Therefore, it is reasonable to assume that all cases where the most recommended drug is not prescribed has a clinical justification. The list of adherence sets together with the reason for which they were added to the list is as follows:

1. $\text{AT}_A = \{\text{ADM}\}$: ADM is the only one of the three oldest TNFis that produces humane antibodies. Physicians who only trust the oldest drugs or are under influence of advertisement by the producer of ADM might deviate from the LIS recommendation to prescribe this drug.
2. $\text{AT}_C = \{\text{CZP}\}$: There have been doubts about the safety of TNFis other than CZP for pregnant persons.
3. $\text{AT}_E = \{\text{ETN}\}$: The half-time of ETN is shorter than that of the other TNFis, and this could be preferable for patients with infection tendencies. It has also shown superior results in monotherapy and is therefore a better alternative than the other four in cases where co-medication should be avoided due to adverse effects of conventional D-MARDs.
4. $\text{AT}_G = \{\text{GOM}\}$: GOM and ADM have been proven to be effective for ulcerative colitis. GOM can also be administered with relatively infrequent dosing. Therefore, it is the only option for patients with ulcerative colitis who prefer infrequent dosing.
5. $\text{AT}_I = \{\text{INX}\}$: INX is the only TNFi that cannot be administered subcutaneously. It might therefore be the preferred option if the physician sees a need for a tighter follow-up of the treatment administration.
6. $\text{C}_{AE} = \{\text{ADM, ETN}\}$: ADM and ETN are the only alternatives out of the three oldest TNFis that can be administered subcutaneously. These are, therefore, the only options if the physician only trusts the oldest drugs and the patient prefers a subcutaneous route.
7. $\text{C}_{AG} = \{\text{ADM, GOM}\}$: ADM and GOM are the only drugs that are proven effective for ulcerative colitis and are available as subcutaneous injection. Patients with ulcerative colitis that strongly prefer subcutaneous belong to this adherence set.
8. $\text{C}_{AI} = \{\text{ADM, INX}\}$: ADM and INX are the only alternatives out of the three oldest TNFis that are proven effective for Crohn's disease. These are, therefore, the only options if the physician only trusts the oldest drugs and the patient has Crohn's disease.
9. $\text{C}_{CG} = \{\text{CZP, GOM}\}$: GOM and CZP can be administered subcutaneously with relatively infrequent dosing. Therefore, they are the only option for patients who strongly prefer infrequent dosing of subcutaneous injections.
10. $\text{NT}_{CE} = \{\text{ADM, GOM, INX}\}$: ADM, GOM and INX are proven effective for Crohn's disease. This set is, therefore, preferred for patients with Crohn's disease.
11. $\text{NT}_{CG} = \{\text{ADM, ETN, INX}\}$: ADM, ETN and INX are the three oldest TNFis, having entered the market much earlier than CZP and GOM. Physicians who only trust the oldest drugs might only choose from this set.

12. NT_{EI} = {ADM, CZP, GOM}: ADM, CZP and GOM produce humane antibodies in contrast to INX and ETN. If humane antibody is important, the physician should choose among these drugs.
13. NT_{I} = {ADM, CZP, ETN, GOM}: INX can not be administered subcutaneously and is known to result in higher levels of antibodies than the other four. Patients who strongly prefer subcutaneous route can only receive one of the other four.
14. C = {ADM, CZP, ETN, GOM, INX}: If none of the reasons above apply in a case, the decision team should be open to all TNFis and comply fully with the LIS recommendation.

A.5 Mathematical expression of the estimators

Let Y be a binary variable indicating remission at three months, $X = \{X_1 - X_{56}\}$ be all the observed confounding factors and T be the treatment where $supp(T)$ = {ADM, CZP, ETN, GOM, INX}. Then our proposed model in the regression analysis is:

$$E(Y|T,X) = P(Y=1|T,X) = (1+\exp(\beta_0 + \sum_{t\in supp(T)} \tau_t \cdot 1[T=t] + \sum_{i=1}^{56} \beta_i X_i))^{-1}$$
$$E(Y^{T=t} - Y^{T=t'}) = \int_{supp(X)} (E(Y|T=t, X=x) - E(Y|T=t', X=x))dp(x),$$

where the second equation assumes no unobserved confounding effects.

Let Z be a vector of treatment alternatives sorted according to the LIS recommendation. For example, in the first LIS year, which is 2010 in our data set, $z_1[1]$ and $z_1[5]$ are the most and the least recommended TNFis by the LIS, respectively. Following the notation used by Kazemi and Olsen [9], for any treatment, $t \in supp(T)$:

$$P_Z(t) : 14 \times 1, P_Z(t)[i] = P(T=t|Z=z_i)$$
$$Q_Z(t) : 14 \times 1, Q_Z(t)[i] = E(Y|Z=z_i, T=t)P(T=t|Z=z_i)$$
$$B_t : 14 \times 14, B_t[i,n] = 1[T=t|Z=z_i, A=a_n],$$

where the indices i and n indicate the LIS year and the adherence set, respectively, both ranging from 1 to 14. For any pair of treatments, (t, t'), let $b_{t-t'}$ be a binary vector satisfying $b_{t-t'} = b_{t-t'}B_t^+ B_t = b_{t-t'}B_{t'}^+ B_{t'}$, where B_t^+ is the Moore–Penrose pseudo-inverse of B_t. Then, $\Pi_{t-t'} = \{a|b_{t-t'}[a] = 1\}$ is the set of adherence sets for which the effect of t vs t' is identified, i.e. Kazemi and Olsen [9] show that:

$$E(Y^{T=t} - Y^{T=t'}|A \in \Pi_{t-t'}) = \frac{b_{t-t'}B_t^+ Q_Z(t)}{b_{t-t'}B_t^+ P_Z(t)} - \frac{b_{t-t'}B_{t'}^+ Q_Z(t')}{b_{t-t'}B_{t'}^+ P_Z(t')}.$$

This is the proposed IV model, which identifies the LATE. To generalize the LATE to the entire population, we also need to assume homogeneity, meaning:

$$E(Y^{T=t} - Y^{T=t'}|A \in \Pi_{t-t'}) = E(Y^{T=t} - Y^{T=t'}).$$

Treatment	ADM (432)	CZP (295)	ETN (472)	GOM (65)	INX (182)	All (1446)
Missingness (%)	11	17	43	80	26	28
Hypertension	16	22	21	8	17	19
Angina Pectoris	0	1	2	0	0	1
Myocardial Infarction	2	3	2	0	2	2
Heart Failure	1	0	1	0	0	0
PCI/CABG	1	3	2	0	3	2
Peripheral artery disease	1	2	1	0	1	1
CH, IS or a TIA	2	1	2	8	3	2
Premature familiar CVD	9	14	14	23	11	12
Pulmonary disease	13	21	22	23	10	17
Allergy/hay-fever/eczema	19	20	28	15	16	21
Chronic low back pain	8	13	12	15	9	10
Cancer	3	2	5	8	1	3
Neurological disease	1	2	2	0	2	2
Diabetes	3	6	6	0	1	4
Hypothyroidism	11	11	12	0	7	11
Mental disease	5	2	6	0	2	4
Alcohol or drugs problem	0	0	1	0	1	0
Kidney disease	1	3	2	0	1	2
Liver disease	1	1	1	0	0	1
Stomach disease	5	11	7	0	5	7
Blood disease	3	2	4	0	2	3

Table B1 Percentage of patients with co-morbidities. In the heading, the number of patients in each treatment group are shown in the parenthesis. The first row indicates the percentage of missing comorbidity data. Abbreviations: PCI: percutaneous coronary intervention, CABG: coronary artery bypass grafting, CH: cerebral hemorrhage, IS: ischemic stroke, TIA: transient ischemic attack, CVD: cardiovascular disease.

The results of our subgroup analyses can be utilized to evaluate this assumption. If homogeneity is violated, the IV results are valid only for the subpopulation for which they are identified, i.e. where at least both treatments compared are relevant alternatives.

Appendix B Further Analyses

B.1 Co-morbidities

The regression analysis is, in addition to baseline variables listed in Subsection A.3, adjusted for 21 co-morbidity variables, measured at baseline. These are listed in Table B1 together with their frequency and missingness statistics.

Contrast	Estimate	CI
CZP - ADM	-0.08	(-0.23,0.08)
ETN - ADM	-0.07	(-0.21,0.08)
ETN - CZP	0.11	(-0.06,0.28)
GOM - CZP	-0.22	(-0.48,0.04)
INX - CZP	0.44	(-0.01,0.91)
GOM - ETN	-0.27	(-0.61,0.02)
INX - ETN	0.39	(-0.22,0.91)

Table B2 The effect estimates from the IV analyses together with confidence interval, using Π_3 as the set of adherence sets.

B.2 Secondary Analysis Requiring Identifiability

In our primary IV analysis, no contrasts are identified involving ADM. Therefore, a secondary IV analysis is performed, with different assumptions on the existence of adherence sets, to inform comparative efficacy of ADM.

For any treatment t, let the degree of t, $d(t)$, be the number of head-to-head comparisons t can be a part of with $\hat{P}(A \in \Pi_{t-t'}) > 0.1$. The list of adherence sets is optimized in the following way:

1. Start with the set presented in Subsection A.4 and call it Π_1
2. Find the largest set Π_2 such that $\{\text{NT}_I, \text{C}\} \subseteq \Pi_2 \subseteq \Pi_1$ and $d(t) > 1 \ \forall \ t$.
3. Find the largest set Π_3 such that $\Pi_2 \subseteq \Pi_3$ and $d(t) > 1 \ \forall \ t$.

From a clinical point of view, NT_I and C are the most important adherence sets. Therefore, we require these two sets to be a part of Π_2 and Π_3 in step 2.

The result of this algorithm is shown in Equation B1. To achieve minimum degree of two, adherence sets $9 - 12$ must be replaced with four new sets, presented below as $15 - 18$, :

15. $\text{C}_{CE} = \{\text{CZP, ETN}\}$
16. $\text{C}_{GI} = \{\text{GOM, INX}\}$
17. $\text{C}_{EG} = \{\text{ETN, GOM}\}$
18. $\text{NT}_G = \{\text{ADM, CZP, ETN, INX}\}$

$$\Pi_3 = (\Pi_1 \backslash \{\text{C}_{CG}, \text{NT}_{CE}, \text{NT}_{CG}, \text{NT}_{EI}\}) \ \cup \ \{\text{C}_{CE}, \text{C}_{GI}, \text{C}_{EG}, \text{NT}_G\} \tag{B1}$$

The new identifiable effects are shown in Figure B3. The results of the comparisons in this figure are presented in Table B2. The results show no difference in the efficacy of ADM vs CZP or ETN. The point estimates are slightly negative, placing ADM somewhere between ETN and INX.

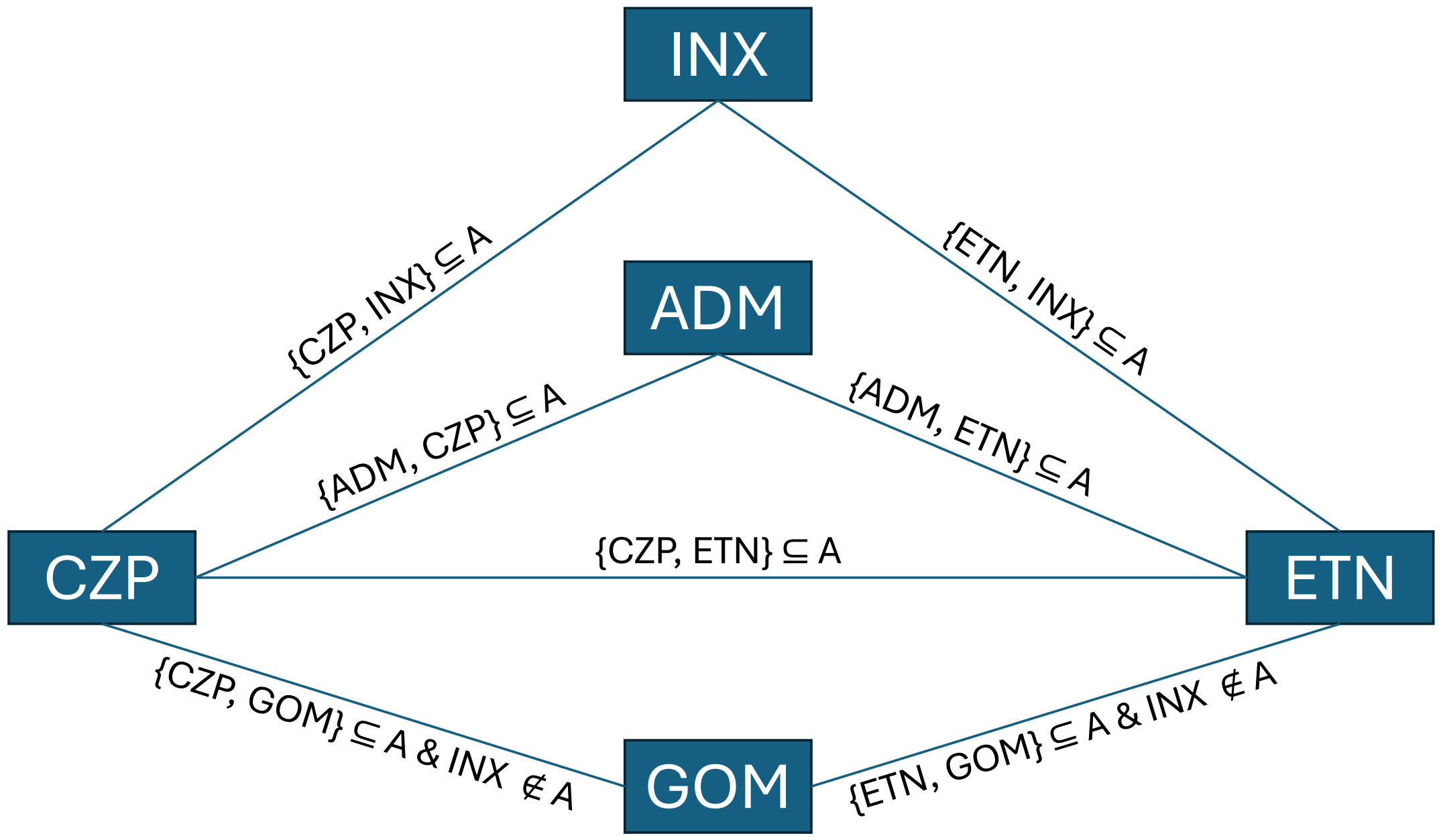

Fig. B3 An edge between two treatments means an identifiable effect. On each edge the condition of the identifiable LATE is provided ($\Pi_{t-t'}$). The conditions are on the adherence sets (A) of the decision teams in the target populations. In this analysis A can only belong to Π_3 as specified in Equaion B1. Abbreviations: CZP: certolizumab pegol, INX: infliximab, GOM: golimumab, ETN: etanercept, ADM: adalimumab. **Alt text:** A presentation of the head-to-head comparisons in the secondary instrumental variable analysis, showing that CZP and ETN are compared to each other and the three other drugs, while INX, ADM and GOM are not directly compared to each other.

B.3 Sensitivity Analysis with Full List of Adherence Sets

In the primary IV analysis, we assume all adherence sets not listed in Subsection A.4 have zero probabilities. As a sensitivity analysis, this assumption is relaxed, letting all possible adherence sets have non-zero probabilities. The complete list of adherence sets, Π_f, is expressed in Equation B2. With this relaxed assumption, the ETN−GOM effect is not identifiable. The identifiable effects are shown in Figure B4. The results of the comparisons in this figure are presented in Table B2. This sensitivity analysis confirms the results from the primary IV analysis.

$$\Pi_f = \{a|a \subseteq C \ \& \ a \neq \emptyset\}, \tag{B2}$$

where $C = \{\text{ADM, CZP, ETN, GOM, INX}\}$.

B.4 Sensitivity Analysis Adjusting for Baseline Variables

We perform a sensitivity IV analysis adjusted for center, sex, age, smoking status, anti-CCP positivity, diagnosis duration, symptom duration, previous MTX use, current MTX use, SJC28, TJC28, PGA,

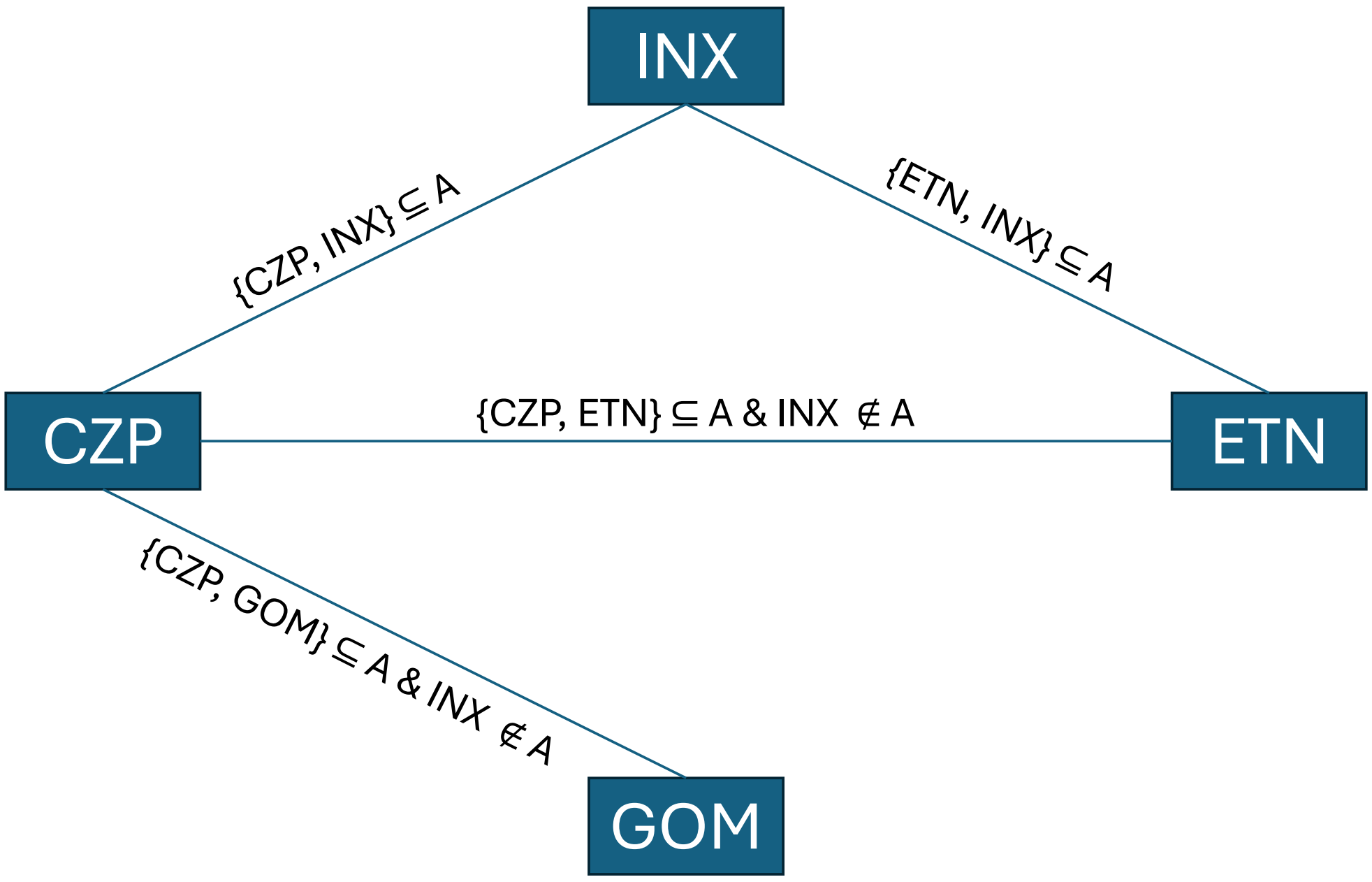

Fig. B4 An edge between two treatments means an identifiable effect. On each edge the condition of the identifiable LATE is provided($\Pi_{t-t'}$). The conditions are on the adherence sets (A) of the decision teams in the target populations. In this analysis A can only belong to Π_f as defined in Equation B2. Abbreviations: CZP: certolizumab pegol, INX: infliximab, GOM: golimumab, ETN: etanercept, ADM: adalimumab. **Alt text:** A presentation of the Head-to-head comparisons in the sensitivity instrumental variable analysis, showing that ADM is not part of any comparisons, CZP is compared to INX, ETN and GOM. ETN is additionally compared to INX.

Contrast	Estimate	CI
ETN - CZP	0.03	(-0.16,0.22)
GOM - CZP	-0.22	(-0.48,0.04)
INX - CZP	0.45	(0,0.91)
INX - ETN	0.37	(-0.25,0.9)

Table B3 The effect estimates from the IV analyses together with confidence interval, using Π_f as the set of adherence sets.

CRP and ESR at baseline. The results are presented in Table B4. These results agree with the results from the primary IV analysis.

B.5 Sensitivity Analysis with Single Imputation of the Outcome

A sensitivity analysis is performed where missing outcome is imputed based on DAS28 ESR, CDAI and termination date in that order. For patients with missing DAS28 CRP, DAS28 ESR and CDAI, the

Contrast	Estimate	CI
ETN - CZP	0.02	(-0.19,0.21)
GOM - CZP	-0.2	(-0.44,0.10)
GOM - ETN	-0.26	(-0.47,0.02)
INX - CZP	0.53	(0.06,0.95)
INX - ETN	0.25	(-0.38,0.88)

Table B4 The effect estimates from the IV analyses together with confidence interval, adjusted for baseline variables.

outcome is set to non-remission if the treatment is discontinued less than a year after baseline. Otherwise, the outcome is set to remission. Missingness in other variables is still handled with MICE using $m = 10$. Our methods of estimating uncertainty, explained in Subsection A.1, also remain unchanged. The results are presented in Figure B5. In this sensitivity analysis, the IV estimates converge towards the null, rendering previous significant results, non-significant. However, the estimates still point in the same direction. For the regression analysis, the opposite phenomenon is observed with estimates moving away from the null, indicating that ADM is significantly superior to INX and CZP. However, for both analyses, all confidence intervals overlap substantially with the confidence intervals from the primary analysis. Given the overlapping confidence intervals and that the analyses are exploratory and not adjusted for multiple comparisons, we conclude that while the significance of our results might be sensitive to the method of handling missing data, this sensitivity analysis is generally consistent with our conclusions.

B.6 Subgroup Analyses

We perform two subgroup analysis, i.e. one in which the analysis is stratified on age and another in which it is stratified on seropositivity. The results of these analyses are presented in Tables B5 and B6, respectively. This analysis indicates that INX's superiority over ETN is strongest among the younger and seropositive patients. In addition, ETN's and CZP's superiority to GOM are greater for older patients and INX's superiority to CZP is strongest for seronegative patients. The result of this analysis does not support homogeneity of the effects.

B.7 Estimated Summary of Baseline Variables

For each comparison in the IV analysis the summary of baseline variables in the corresponding target population is estimated and presented in Table B7.

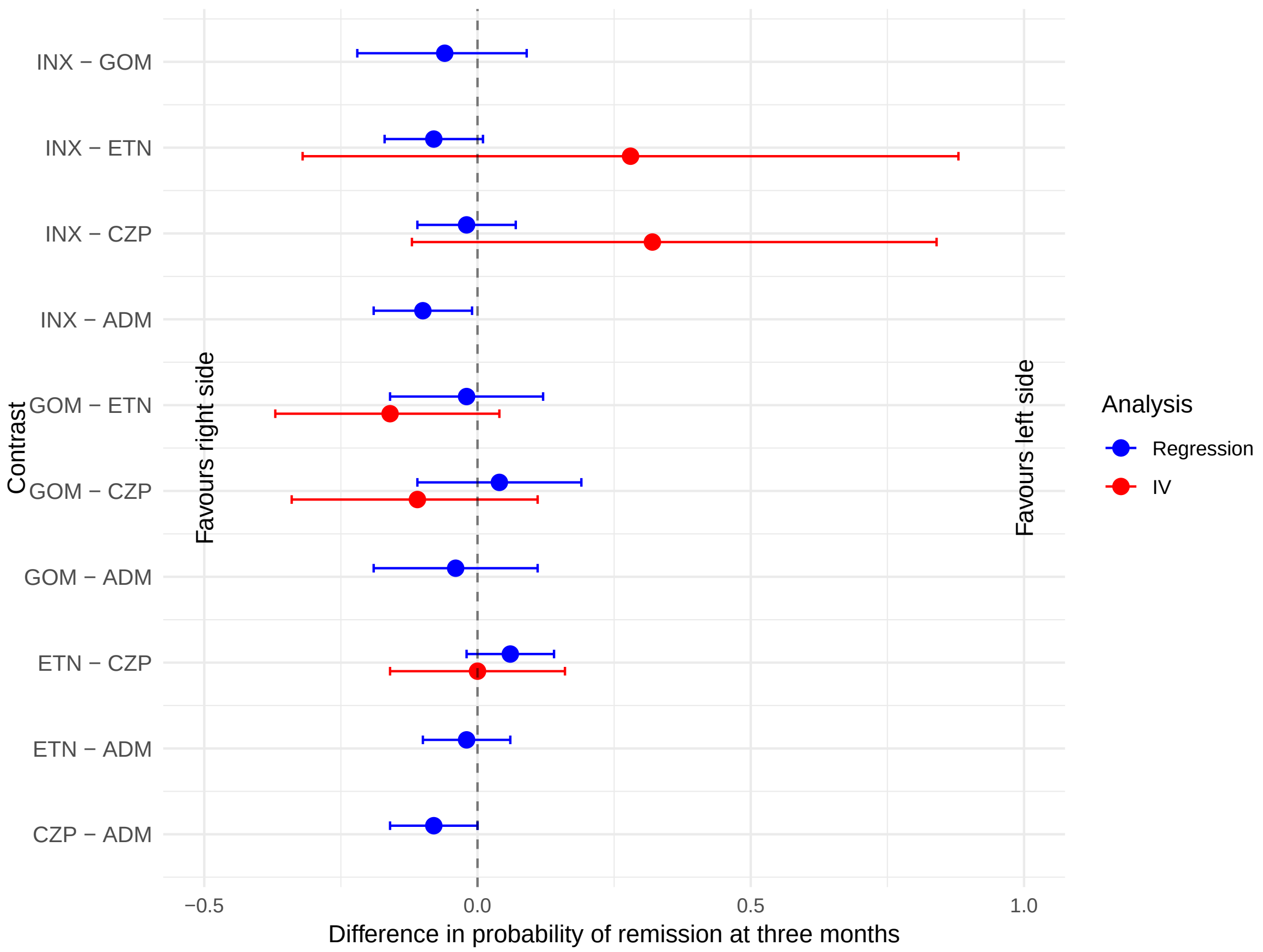

Fig. B5 For the regression analysis the CIs are derived analytically from the model while for the IV analysis the CIs are calculated using bootstrapping with 1000 replications, excluding the replications that yield an estimate outside the $[-1, 1]$ interval. In both cases, the uncertainty is calculated for each imputed data set and then pooled. Note that for each contrast, $t_1 - t_2$, the X-axis is $E(Y^{T=t_1} - Y^{T=t_2})$. Therefore, an estimate on the right side of the dashed black line means that the left side of the contrast $t_1 - t_2$ has better efficacy, and vice versa. Abbreviations: CZP: certolizumab pegol, INX: infliximab, GOM: golimumab, ETN: etanercept, ADM: adalimumab. **Alt text:** The effect estimates from both analyses together with confidence intervals using single imputation as described in Subsection B.5.

Appendix C Further Discussions

C.1 Extended background

Ioannidis et al. [4] criticize the research community for the lack of head-to-head randomized trials of biologic drugs in rheumatology and argue that this paucity is due to the "complex interface between the goals of regulatory bodies, the pharmaceutical industry and clinical researchers". They argue that trial sponsors are unwilling to cover the expense of a competitor's drug in a head-to-head trial with the risk of showing their own drug's inferiority. Another reason, they argue, could be that strict regulatory requirements and publication bias favoring positive results incentivize placebo-controlled trials over head-to-head comparisons of active agents. TNFis are compared in observational studies. However,

	Youngest half				Oldest half			
	Regression		IV		Regression		IV	
Contrast	Estimate	CI	Estimate	CI	Estimate	CI	Estimate	CI
CZP - ADM	0.02	(-0.12,0.16)			0.02	(-0.1,0.14)		
ETN - ADM	0.01	(-0.13,0.14)			0.06	(-0.04,0.16)		
ETN - CZP	-0.01	(-0.15,0.12)	0.01	(-0.22,0.23)	0.04	(-0.08,0.16)	0.02	(-0.24,0.28)
GOM - ADM	0.01	(-0.22,0.23)			0.01	(-0.23,0.26)		
GOM - CZP	-0.02	(-0.23,0.2)	-0.10	(-0.42,0.23)	0.00	(-0.26,0.25)	-0.39	(-0.7,-0.03)
GOM - ETN	0.00	(-0.21,0.2)	-0.14	(-0.42,0.14)	-0.04	(-0.29,0.21)	-0.44	(-0.73,-0.12)
INX - ADM	0.03	(-0.13,0.19)			0.01	(-0.12,0.14)		
INX - CZP	0.01	(-0.15,0.18)	0.30	(-0.8,0.91)	-0.01	(-0.14,0.13)	0.47	(0.02,0.9)
INX - ETN	0.02	(-0.14,0.19)	0.82	(-0.42,0.97)	-0.05	(-0.18,0.09)	0.01	(-0.68,0.78)
INX - GOM	0.03	(-0.22,0.27)			0.00	(-0.26,0.25)		

Table B5 Regression and IV analyses results stratified on age $\leq$ or $>$ median age (55).

	Seronegative				Seropositive			
	Regression		IV		Regression		IV	
Contrast	Estimate	CI	Estimate	CI	Estimate	CI	Estimate	CI
CZP - ADM	0.03	(-0.13,0.19)			0.01	(-0.1,0.13)		
ETN - ADM	0.05	(-0.13,0.24)			0.02	(-0.07,0.12)		
ETN - CZP	0.03	(-0.15,0.21)	-0.05	(-0.41,0.27)	0.01	(-0.1,0.12)	0.04	(-0.16,0.26)
GOM - ADM	-0.03	(-0.32,0.26)			0.05	(-0.16,0.25)		
GOM - CZP	-0.06	(-0.34,0.22)	-0.32	(-0.79,0.23)	0.03	(-0.17,0.24)	-0.16	(-0.43,0.1)
GOM - ETN	-0.09	(-0.38,0.21)	-0.26	(-0.63,0.2)	0.02	(-0.19,0.23)	-0.25	(-0.51,-0.01)
INX - ADM	0.05	(-0.14,0.23)			0.01	(-0.11,0.12)		
INX - CZP	0.02	(-0.19,0.22)	0.61	(-0.69,0.95)	-0.01	(-0.14,0.12)	0.41	(-0.14,0.9)
INX - ETN	-0.01	(-0.24,0.23)	-0.16	(-0.91,0.77)	-0.02	(-0.13,0.09)	0.69	(-0.17,0.96)
INX - GOM	0.08	(-0.22,0.38)			-0.04	(-0.26,0.18)		

Table B6 Regression and IV analyses results stratified on seropositivity (anti-CCP $\geq$ 20 u/mL).

study heterogeneity prevents proper network meta-analyses [7]. Sparks et al. [7] call for further research to identify subgroups that may benefit more from specific drugs.

C.2 Justification of Exogeneity

One can argue for the exogeneity of this instrument conceptually. Exogeneity means that the only causal path from the LIS recommendation to clinical remission is through the prescribed treatment. A possible violation is if biosimilar drugs (defined in Appendix Subsection A.2) differ from the original drug in efficacy or population characteristics affecting remission. Patent expiration has a strong effect on price, affecting the LIS recommendation. Therefore, a causal path between the drug producer and the outcome, which is not mediated through the treatment itself, would violate exogeneity. However, previous literature suggests this is unlikely [21].

Treatment	CZP	ETN	CZP	GOM	CZP	INX
Age (years)	53 (50, 57)	50 (47, 54)	55 (52, 58)	50 (45, 55)	58 (45, 72)	56 (53, 60)
Male (%)	43 (32, 55)	34 (24, 44)	41 (32, 52)	28 (11, 46)	24 (1, 62)	31 (20, 42)
Smoker current (%)	18 (9, 30)	16 (8, 27)	16 (7, 25)	31 (14, 50)	35 (6, 78)	22 (9, 33)
Smoker occasionally (%)	1 (0, 5)	2 (0, 6)	2 (0, 5)	3 (0, 11)	4 (0, 22)	2 (0, 9)
Smoker previously (%)	0 (0, 51)	0 (0, 51)	38 (0, 51)	21 (0, 41)	0 (0, 81)	0 (0, 48)
RF positive (%)	46 (31, 60)	62 (50, 73)	51 (39, 63)	79 (61, 94)	70 (24, 98)	51 (37, 63)
Anti-CCP positive (%)	64 (51, 78)	69 (57, 80)	65 (53, 76)	81 (64, 95)	71 (23, 99)	66 (52, 80)
Previous MTX user (%)	98 (91, 100)	81 (72, 89)	92 (85, 99)	71 (53, 88)	95 (76, 100)	90 (83, 99)
MTX at BL (%)	72 (61, 83)	63 (52, 73)	68 (59, 78)	83 (67, 96)	96 (87, 99)	83 (72, 97)
Other DMARDs at BL (%)	53 (41, 65)	41 (29, 51)	54 (43, 64)	85 (69, 98)	68 (26, 98)	56 (45, 70)
DAS28 CRP at BL	4 (3, 4)	4 (3, 4)	4 (3, 4)	4 (4, 5)	4 (3, 5)	4 (4, 4)
Diagnosis duration (years)	7 (4, 9)	5 (4, 7)	9 (7, 11)	6 (4, 9)	11 (3, 23)	6 (4, 9)
Treatment	ETN	GOM	ETN	INX		
Age (years)	50 (47, 54)	50 (45, 54)	60 (44, 87)	54 (51, 57)		
Male (%)	34 (24, 44)	28 (14, 44)	10 (0, 44)	33 (23, 44)		
Smoker current (%)	16 (8, 27)	28 (14, 44)	45 (6, 92)	17 (5, 28)		
Smoker occationally (%)	2 (0, 6)	3 (0, 10)	16 (1, 50)	2 (0, 8)		
Smoker previously (%)	35 (0, 52)	17 (0, 38)	0 (0, 77)	0 (0, 45)		
RF positive (%)	62 (50, 73)	77 (62, 91)	83 (39, 99)	46 (32, 59)		
Anti-CCP positive (%)	69 (57, 80)	79 (65, 93)	88 (47, 100)	65 (53, 76)		
Previous MTX user (%)	81 (72, 89)	71 (56, 86)	15 (0, 49)	92 (85, 99)		
MTX at BL (%)	63 (52, 73)	83 (69, 95)	90 (54, 100)	74 (64, 83)		
Other DMARDs at BL (%)	41 (29, 51)	80 (66, 92)	94 (92, 97)	39 (26, 50)		
DAS28 CRP at BL	4 (3, 4)	4 (4, 5)	5 (4, 8)	4 (4, 4)		
Diagnosis duration (years)	5 (4, 7)	6 (4, 8)	18 (8, 41)	5 (2, 8)		

Table B7 Estimated summary of baseline data for each comparison, stratified on the treatment received. Trials are separated with vertical lines. All values not given in percentage are mean values. Confidence intervals (95%), calculated using multiple imputation with $m = 10$ and bootstrapping with 1000 replications, are provided in parentheses. Abbreviations: BL: Baseline, RF: Rheumatoid factor, CCP: Cyclic citrullinated peptide, MTX: Methotrexate, DMARD: Disease-modifying antirheumatic drug, DAS: Disease activity score, CRP: C-reactive protein.

Exogeneity implies that patient populations from different years are exchangeable, except for varying LIS recommendations. However, one could suspect a spurious correlation of both the instrument and the outcome with time. The price of a drug is expected to decrease after patent expiration, improving the drug's chance of winning the LIS tender. In addition, the remission rate is expected to naturally increase over time due to better clinical practices unrelated to the most common TNFi (e.g. lower disease activity at baseline). However, this trend is weak in our data set (0.6% increase in remission rate pr year). The correlation between patent expiration and improved practices is likely due to two time series aligning over a short period by chance, with no causal link, and can be ignored.

C.3 Justification of the Comparator Method

Regressions analysis has previously been applied to TNFi comparisons in observational studies [7]. We have chosen standardization over inverse probability weighting as the main *conventional* method because we deem the risk of misspecifying the model to be lower for the outcome than it is for the treatment. There are potentially large unobserved confounding effects in our analysis, such as planned pregnancy, unobserved co-morbidities, degree of participation in work, etc. While these unobserved factors may substantially affect the choice of treatment, their effect on the outcome might be smaller. Furthermore, the outcome, as a binary variable, requires fewer assumptions to model than the treatment, which is a categorical variable.

By measuring confounding effects at baseline and the outcome after three months, we limit bias due to time-varying effects. However, unobserved confounders like pregnancy, work status, physician preferences, or co-morbidities such as ulcerative colitis or Crohn's disease may introduce significant bias, resulting in the regression analysis not providing unbiased causal estimates [22, 23].

We avoid doubly robust estimation because despite Uysal [24] and [25] encouraging such a methodology in the case of categorical treatment, such methods are not yet well established and well tested in epidemiological research. Additionally, one could argue that in a simple setting like ours, in the absence of time-varying variables and potential colliders, the increased robustness of these methods might not be worth the decreased efficiency and transparency.

C.4 Examples of Unobserved Confounding Effects

ETN might be more frequently prescribed to patients with infection tendencies or other co-morbidities that make its short half-time favorable. CZP might be more frequently prescribed to patients who plan pregnancy. INX, GOM and ADM have been proven effective for Crohn's disease and might be more frequently prescribed to these patients. INX is more likely to be prescribed to patients that are not fully employed and therefore do not favour subcutaneous administration. All of these factors can plausibly affect the outcome.

References

[1] Smolen, J.S., Aletaha, D., McInnes, I.B.: Rheumatoid arthritis. The Lancet **388**(10055), 2023–2038 (2016) https://doi.org/10.1016/S0140-6736(16)30173-8 . Erratum in: The Lancet, 2016 Oct 22;388(10055):1984. doi: 10.1016/S0140-6736(16)30794-2. Published online 2016 May 3.

[2] Kerschbaumer, A., Sepriano, A., Bergstra, S.A., Smolen, J.S., Van Der Heijde, D., Caporali, R., Edwards, C.J., Verschueren, P., De Souza, S., Pope, J.E., *et al.*: Efficacy of synthetic and biological dmards: a systematic literature review informing the 2022 update of the eular recommendations for the management of rheumatoid arthritis. Annals of the rheumatic diseases **82**(1), 95–106 (2023)

[3] Gehin, J.E., Goll, G.L., Brun, M.K., Jani, M., Bolstad, N., Syversen, S.W.: Assessing immunogenicity of biologic drugs in inflammatory joint diseases: progress towards personalized medicine. BioDrugs **36**(6), 731–748 (2022)

[4] Ioannidis, J.P., Karassa, F.B., Druyts, E., Thorlund, K., Mills, E.J.: Biologic agents in rheumatology: unmet issues after 200 trials and $200 billionsales.NatureReviewsRheumatology\mathbf{9}(11), 665 - -673(2013)$

[5] Kerschbaumer, A., Sepriano, A., Smolen, J.S., Heijde, D., Dougados, M., Vollenhoven, R., McInnes, I.B., Bijlsma, J.W., Burmester, G.R., Wit, M., *et al.*: Efficacy of pharmacological treatment in rheumatoid arthritis: a systematic literature research informing the 2019 update of the eular recommendations for management of rheumatoid arthritis. Annals of the rheumatic diseases **79**(6), 744–759 (2020)

[6] Smolen, J.S., Burmester, G.-R., Combe, B., Curtis, J.R., Hall, S., Haraoui, B., Vollenhoven, R., Cioffi, C., Ecoffet, C., Gervitz, L., *et al.*: Head-to-head comparison of certolizumab pegol versus adalimumab in rheumatoid arthritis: 2-year efficacy and safety results from the randomised exxelerate study. The Lancet **388**(10061), 2763–2774 (2016)

[7] Sparks, J.A., Harrold, L.R., Simon, T.A., Wittstock, K., Kelly, S., Lozenski, K., Khaychuk, V., Michaud, K.: Comparative effectiveness of treatments for rheumatoid arthritis in clinical practice: A systematic review. In: Seminars in Arthritis and Rheumatism, p. 152249 (2023). Elsevier

[8] Mielnik, P.: Drug procurement cooperation (lis)-norwegian tender system to reduce cost of expensive medicines. Journal of Health Policy and Outcomes Research (1), 18–31 (2014)

[9] Kazemi, A.A., Olsen, I.C.: Instrumental variable analysis with categorical treatment. Statistical Methods in Medical Research **33**(11-12), 2043–2061 (2024)

[10] Greenland, S.: Estimating standardized parameters from generalized linear models. Statistics in

medicine **10**(7), 1069–1074 (1991)

[11] Gharibzadeh, S., Mohammad, K., Rahimiforoushani, A., Amouzegar, A., Mansournia, M.A.: Standardization as a tool for causal inference in medical research. Archives of Iranian medicine **19**(9), 0–0 (2016)

[12] Smolen, J.S., Landewé, R.B., Bergstra, S.A., Kerschbaumer, A., Sepriano, A., Aletaha, D., Caporali, R., Edwards, C.J., Hyrich, K.L., Pope, J.E., *et al.*: Eular recommendations for the management of rheumatoid arthritis with synthetic and biological disease-modifying antirheumatic drugs: 2022 update. Annals of the rheumatic diseases **82**(1), 3–18 (2023)

[13] Heckman, J.J., Vytlacil, E.: Policy-relevant treatment effects. American Economic Review **91**(2), 107–111 (2001)

[14] White, I.R., Royston, P., Wood, A.M.: Multiple imputation using chained equations: issues and guidance for practice. Statistics in medicine **30**(4), 377–399 (2011)

[15] Azur, M.J., Stuart, E.A., Frangakis, C., Leaf, P.J.: Multiple imputation by chained equations: what is it and how does it work? International journal of methods in psychiatric research **20**(1), 40–49 (2011)

[16] Schomaker, M., Heumann, C.: Bootstrap inference when using multiple imputation. Statistics in medicine **37**(14), 2252–2266 (2018)

[17] Olsen, I., Kvien, T., Uhlig, T.: Consequences of handling missing data for treatment response in osteoarthritis: a simulation study. Osteoarthritis and cartilage **20**(8), 822–828 (2012)

[18] Pan, H., Peto, R., Restrepo, A.M.H., Preziosi, M.-P., Sathiyamoorthy, V., Karim, Q.A., Alejandria, M., García, C.H., Kieny, M.-P., Malekzadeh, R., *et al.*: Remdesivir and three other drugs for hospitalised patients with covid-19: final results of the who solidarity randomised trial and updated meta-analyses. The Lancet **399**(10339), 1941–1953 (2022)

[19] Normand, S.-L.T.: The RECOVERY platform. Mass Medical Soc (2021)

[20] Van Buuren, S., Groothuis-Oudshoorn, K.: mice: Multivariate imputation by chained equations in r. Journal of statistical software **45**, 1–67 (2011)

[21] Oliveira Ascef, B., Almeida, M.O., Medeiros-Ribeiro, A.C., Andrade, D.C.O., Oliveira Junior, H.A., Soarez, P.C.: Therapeutic equivalence of biosimilar and reference biologic drugs in rheumatoid arthritis: A systematic review and meta-analysis. JAMA Network Open **6**(5), 2315872–2315872 (2023)

[22] Gordon, H., Minozzi, S., Kopylov, U., Verstockt, B., Chaparro, M., Buskens, C., Warusavitarne, J., Agrawal, M., Allocca, M., Atreya, R., Battat, R., Bettenworth, D., Bislenghi, G., Brown, S.R., Burisch, J., Casanova, M.J., Czuber-Dochan, W., Groof, J., El-Hussuna, A., Ellul, P., Fidalgo, C., Fiorino, G., Gisbert, J.P., Sabino, J.G., Hanzel, J., Holubar, S., Iacucci, M., Iqbal, N., Kapizioni, C., Karmiris, K., Kobayashi, T., Kotze, P.G., Luglio, G., Maaser, C., Moran, G., Noor, N., Papamichael, K., Peros, G., Reenaers, C., Sica, G., Sigall-Boneh, R., Vavricka, S.R., Yanai, H., Myrelid, P., Adamina, M., Raine, T.: Ecco guidelines on therapeutics in crohn's disease: Medical treatment. Journal of Crohn's and Colitis **18**(10), 1531–1555 (2024) https://doi.org/10.1093/ecco-jcc/jjae091 https://academic.oup.com/ecco-jcc/article-pdf/18/10/1531/59765621/jjae091.pdf

[23] Le Berre, C., Honap, S., Peyrin-Biroulet, L.: Ulcerative colitis. The Lancet **402**(10401), 571–584 (2023)

[24] Uysal, S.D.: Doubly robust estimation of causal effects with multivalued treatments: an application to the returns to schooling. Journal of Applied Econometrics **30**(5), 763–786 (2015)

[25] Linden, A., Uysal, S.D., Ryan, A., Adams, J.L.: Estimating causal effects for multivalued treatments: a comparison of approaches. Statistics in Medicine **35**(4), 534–552 (2016)
